# High-altitude and low-altitude adapted chicken gut-microbes have different functional diversity

**DOI:** 10.1038/s41598-023-48147-9

**Published:** 2023-11-27

**Authors:** Neha Rani Bhagat, Priyanka Chauhan, Pratibha Verma, Aradhana Mishra, Vijay K. Bharti

**Affiliations:** 1 DRDO-Defence Institute of High-Altitude Research (DIHAR), Ministry of Defence, Leh, 194101 UT Ladakh India; 2 Department of Microbiology, Panjab University, Chandigarh, India; 3 Academy of Scientific and Innovative Research (AcSIR), Ghaziabad, 201002 India; 4 School of Sciences, P. P. Savani University, NH-8, GETCO, Near Biltech, Kosamba, Surat, 394125 India; 5 Division of Microbial Technology, CSIR-National Botanical Research Institute, Lucknow, 226001 Uttar Pradesh India

**Keywords:** Biological techniques, Ecology, Microbiology, Zoology

## Abstract

Recently, there has been considerable interest in the functions of gut microbiota in broiler chickens in relation to their use as feed additives. However, the gut-microbiota of chickens reared at different altitudes are not well documented for their potential role in adapting to prevailing conditions and functional changes. In this context, the present study investigates the functional diversity of gut-microbes in high-altitude (HACh) and low-altitude adapted chickens (LACh), assessing their substrate utilization profile through Biolog Ecoplates technology. This will help in the identification of potential microbes or their synthesized metabolites, which could be beneficial for the host or industrial applications. Results revealed that among the 31 different types of studied substrates, only polymers, carbohydrates, carboxylic acids, and amine-based substrates utilization varied significantly (p < 0.05) among the chickens reared at two different altitudes where gut-microbes of LACh utilized a broad range of substrates than the HACh. Further, diversity indices (Shannon and MacIntosh) analysis in LACh samples showed significant (p < 0.05) higher richness and evenness of microbes as compared to the HACh samples. However, no significant difference was observed in the Simpson diversity index in gut microbes of lowversus high-altitude chickens. In addition, the Principal Component Analysis elucidated variation in substrate preferences of gut-microbes, where 13 and 8 carbon substrates were found to constitute PC1 and PC2, respectively, where γ-aminobutyric acid, d-glucosaminic acid, i-erythritol and tween 40 were the most relevant substrates that had a major effect on PC1, however, alpha-ketobutyric acid and glycyl-l-glutamic acid affected PC2. Hence, this study concludes that the gut-microbes of high and low-altitudes adapted chickens use different carbon substrates so that they could play a vital role in the health and immunity of an animal host based on their geographical location. Consequently, this study substantiates the difference in the substrate utilization and functional diversity of the microbial flora in chickens reared at high and low altitudes due to altitudinal changes.

## Introduction

The gut microbiota is recently known to perform essential biological functions, nevertheless, alterations in its potential are attributed to the host's geographical location (altitude), which may prompt diverse physiological and metabolic changes within the host^1–3^. Similarly, gut microbes are also characterized to have  an essential role in promoting the nutrient absorption, health, growth performance, metabolism, and immunity of broiler chickens, where various factors affect their composition such as  age, diet, genetics, antibiotic feeding, altitude, and environmental conditions^4–6^. Previous studies have also stated that altitude variation could majorly reshape the composition and diversity of gut microbes for adaptation to an environment, especially high altitude by augmenting the host energy and glycan biosynthesis^6,7^. In fact, it has been discovered that various microbial community structures around the world have adopted different enterotypes of microflora to support their survival and functional activities in the relevant geographical location^8^. In this view, High-altitude regions are one of the most challenging terrains, yet the least studied regions of the world, where sustainability of non-native humans and animals becomes difficult during their acute ascent^9,10^.

Moreover, numerous studies have highlighted substantial variations between the gut bacterial community of high and low-altitude inhabitant animals such as pika, mice, rats, Tibetan chickens, etc.^6,11–14^. For instance, In Tibetan chickens, the altitudinal shift has led to an increased abundance of phyla Firmicutes, Bacteroidetes, Actinobacteria, and Proteobacteria at higher altitudes, where phylum Firmicutes was observed to rise with altitude in their ileum^6^. Although the role of gut microbial communities in adaptation, health, immunity, metabolism, and productivity of animals is recognized to be significant for High-altitude regions, the existing research on this topic has primarily focused on bioinformatic analysis to predict their distinctiveness, resilience, and extensive functional capabilities^6,15^. This makes them an ideal candidate for broad biotechnological implications such as producing potential enzymes, biomolecules, metabolites, bioactive compounds, or feed additives. However, there is a lack of in-vitro studies that have investigated the diversity, metabolic patterns, and specific functions of these microbes, with only a limited number of such studies conducted^16^. Therefore, in order to determine gut microbial diversity and its role in metabolism, recently, methods such as Metagenomics, metabolomics, proteomics, and phospholipid fatty acids analysis (PLFA), have become popular. However, the methods afore-mentioned are costly, time-taking, not comprehensive, and restricted due to several limitations, including the ineffective and inconsistent community DNA extraction techniques, differences in the sequencing technologies used to analyze the gut microbiome, as well as their restriction only up to the prediction of physiological and functional properties of the microbial community^17–20^.

In this regard, Biolog EcoPlates is a sensitive and reliable indicator of the microbiological diversity and their functional profile due to environmental change in the samples^21^. This assay involves 31 carbon substrates, categorised as (a) Polymers: Tween 40; Tween 80; α-Cyclodextrin and Glycogen, (b) Carbohydrates: d-Cellobiose; α-d-Lactose; Methyl-d-glucoside; d-Xylose; i-Erythritol; d-Mannitol; *N*-Acetyl-d-glucosamine; d,l-α-Glycerol phosphate; Glucose-1-phosphate; and Pyruvic acid methyl ester, (c) Carboxylic acids: d-Glucosaminic acid, d-Galactonic acid lactone, d-Galacturonic acid, γ-aminobutyric acid, Itaconic acid, α-Keto butyric acid, and d-Malic acid, (d) Amino acids: l-Arginine; l-Asparagine; l-Phenylalanine; l-Serine; l-Threonine; and Glycyl-l-glutamic acid, (e) Amines/amides: Phenylethylamine and, Putrescine, and (f) Phenolic acids: 2-Hydroxy benzoic acid, and 4-Hydroxy benzoic acid. Evaluating the utilisation pattern of these carbon substrates using these Biolog Ecoplates provides a snapshot of the actual metabolism and physiological profiles of microbial diversity in the host and microbial habitats^21^. This knowledge on differential substrate utilization by microbes gives valuable clues on the types of microbes, their secretary molecules, and host-microbes nutrient utilization patterns. Further, this data will also aid in the identification of suitable microbes for developing a novel probiotic formulation, the screening of suitable substrates for the in-vitro *culture* of microbes for their secretary molecules, and elucidating the profile of nutrient supply to the host. This method also has various advantages in distinguishing and determining spatial functional changes in microbial communities^22^. Therefore, in the present study, the metabolic potential based on the substrate’s utilization pattern of gut-microbes from high-altitude (HACh) and low-altitude adapted (LACh) chickens is elucidated using Biolog Ecoplates technology. This will help to analyse the functional diversity of the gut microbial community between high and low altitude Chickens.

## Materials and methods

### Study location

**Figure 1:**
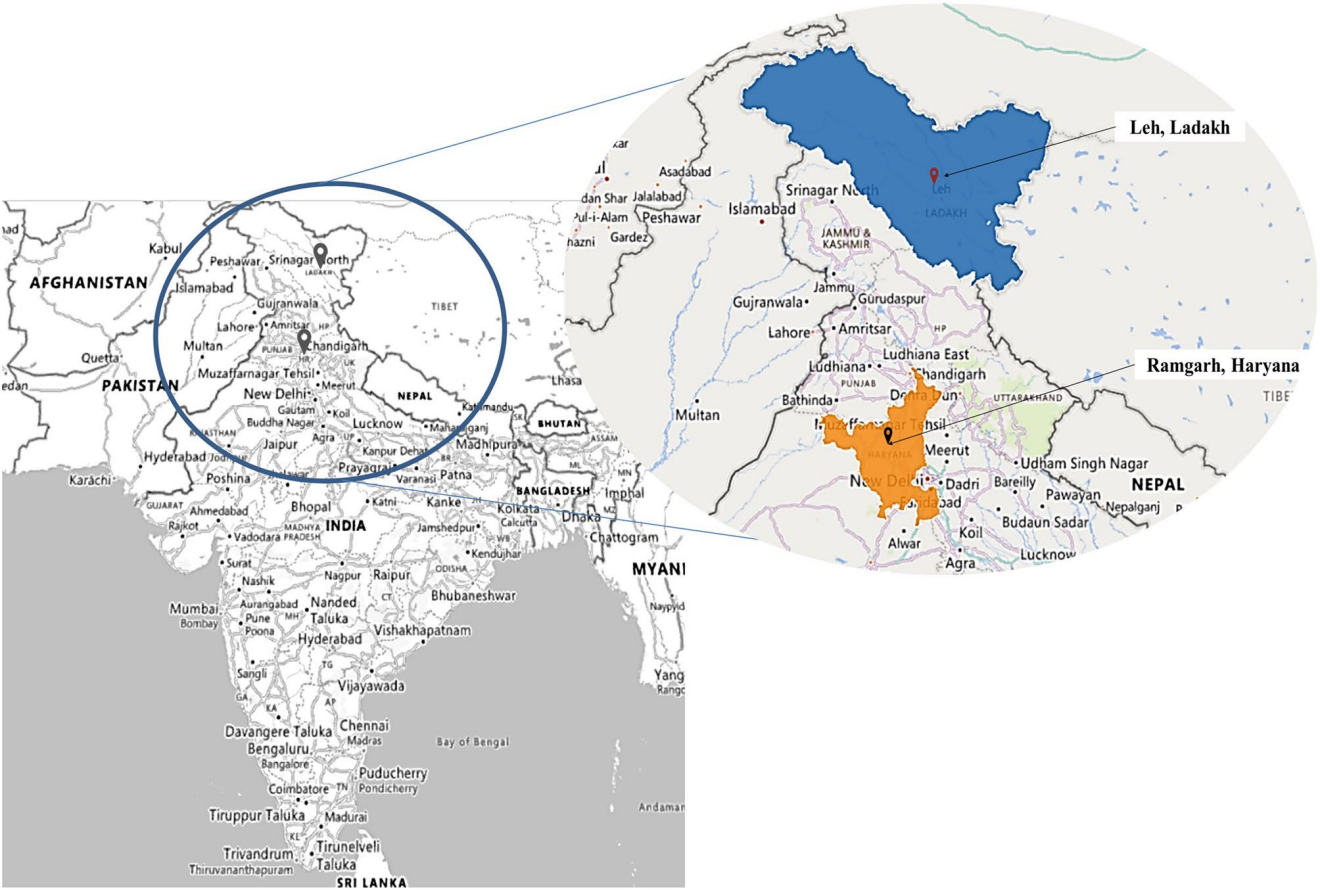
Geographical position of the study locations (Leh, India: High Altitude; Ramgarh, India: Low altitude) (created using MS Excel-3DMaps and MS PowerPoint, Office-Version 2309).

The comparative study was carried out on chickens reared at the Animal Farms of Defense Institute located at a High altitude of 3500 m i.e., Leh-Ladakh (34.152588 N, 77.577049 E), a large town of 45,100 km^2^ and 297,000 inhabitants located in the northern area of Ladakh state, India, whereas, low altitude region of elevation 289 m i.e., Ramgarh (30.648220 N, 76.887230 S), a small village of 3.67 km^2^ and 5644 inhabitants in the state of Haryana, India (Fig. 1). These DIHAR chickens were kept under a deep litter system in the solar poultry house, where the ambient temperature of the house was maintained at 25–27 °C. This housing system overcomes the extreme temperature effect prevalent in high-altitude, Ladakh. Similarly, In Ramgarh, the birds were also housed in a deep litter system with an ambient temperature of the house maintained at 25–29 °C and relative humidity at 65–67%. All chickens were fed as per the standard readymade commercial poultry ration with free access to water.

### Sample collection and preparation

The fecal microbial community mirrors the microbial structure in the intestine at the microbial population level, with phylum specificity in chickens. Therefore, in this study, a total of six Cloacal fecal samples (n = 6) were collected from live, adult chickens of each study location and then immediately transferred to a sterile falcon tube using a sterile swab. These samples were then pooled to prepare two sample groups, i.e., High-altitude chicken (HACh) and Low-altitude chicken (LACh). Thereafter, these pooled samples from each group were mixed with sterile normal saline, left for incubation at Room Temperature (25–27 °C) for an hour, and diluted to give dilutions up to 10^–2^. These prepared samples are then further analysed for substrate utilization using Biolog Ecoplate assay. Notably, Using pooled samples to characterize the metabolic potential and functional diversity of microbial communities is a more cost-efficient, precise, and less biased parameter estimation than that occurs with individual samples. The pooled sample study model is only disadvantageous in associational and longitudinal studies.

### Biolog ecoplate assay

In brief, the substrate utilization by the chicken gut microbiota was assessed using Biolog Ecoplates containing three replicate sets of 31 relevant carbon substrates utilized^23^. Each well of Biolog Ecoplate plate was inoculated with 150 µL of 10^–2^ diluted samples in three replicate sets of each substrate in each group, followed by incubation in oxic conditions for 7 days at 28 ± 2 °C. The absorbance was measured at 590 nm at 24 h interval. Then, the utilization pattern was observed by the reduction of tetrazolium dye, which switches from colourless to purple during the growth of microbes. The results have been expressed as average well colour development (AWCD) = ΣODi/31^22,24,25^. Mean optical density for the time-dependant utilization pattern of each substrate as well as different diversity indices for heterogeneity i.e., richness and evenness of microbes for functional differences in samples. Thereafter, the optical density of 96 h incubation time was used to calculate the statistical difference in utilization of individual substrates and diversity indices as previously described^26^.

### Statistical analyses

Paired t-test was performed in SPSS version 24.0 to compare the substrate utilisation between the animal groups. Moreover, Diversity indices and Multivariate Principal Component Analysis (PCA) were performed to evaluate the distribution of substrate utilization in EcoPlates using the PAST 4.03 software.

### Limitation

This investigation fails to study the unculturable microbes in lab conditions and concerns only culturable microorganisms for substrate utilization and their metabolic potential, which is the main objective of the study.

## Results

### Average well colour development

**Figure 2:**
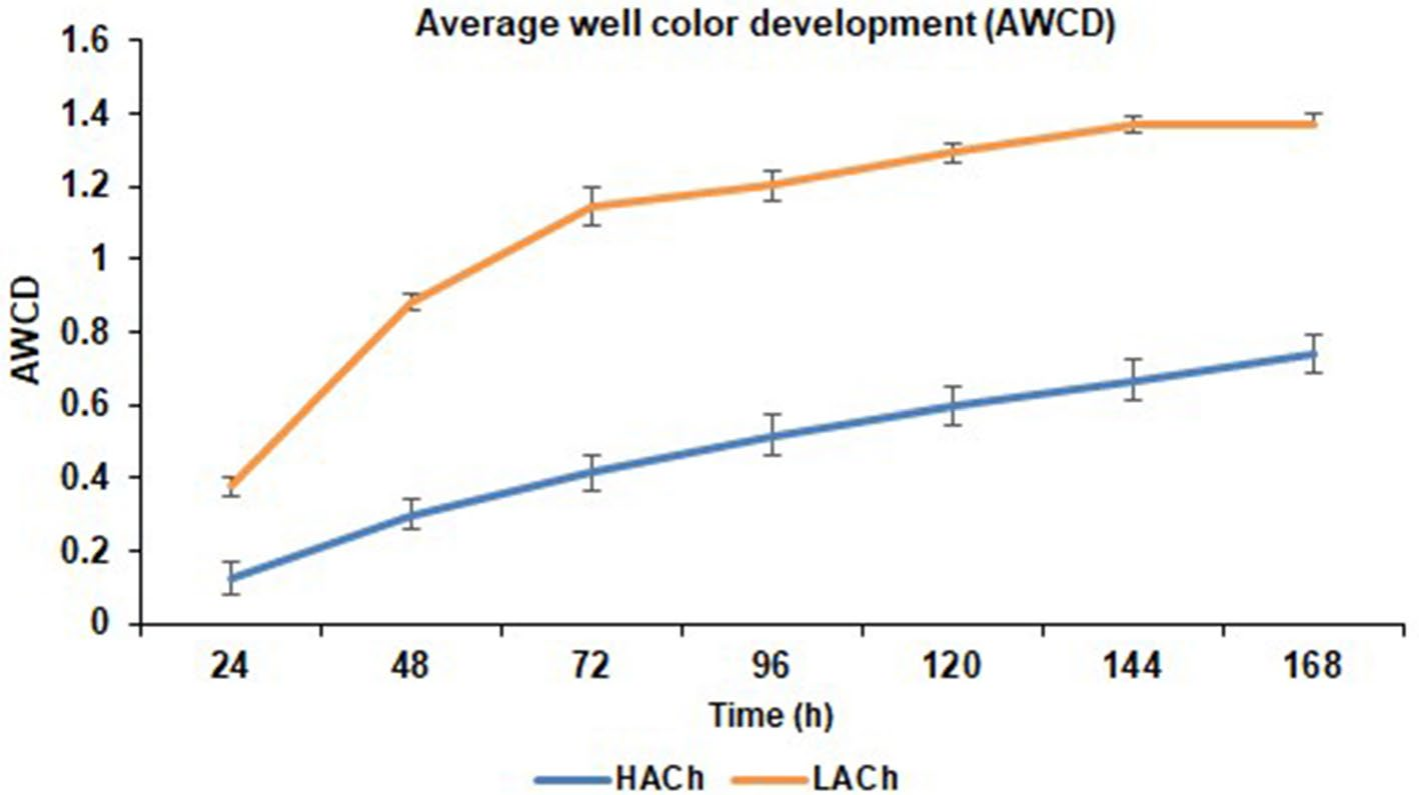
Average well colour development (AWCD) at different incubation times.

To test the metabolic activity of gut-microbes in both samples collected from LACh and HACh, overall Average Well Color Development (AWCD) over 168 h, revealed a significantly (p < 0.05) higher metabolic activity in LACh as compared to HACh (Fig. 2).

**Figure 3:**
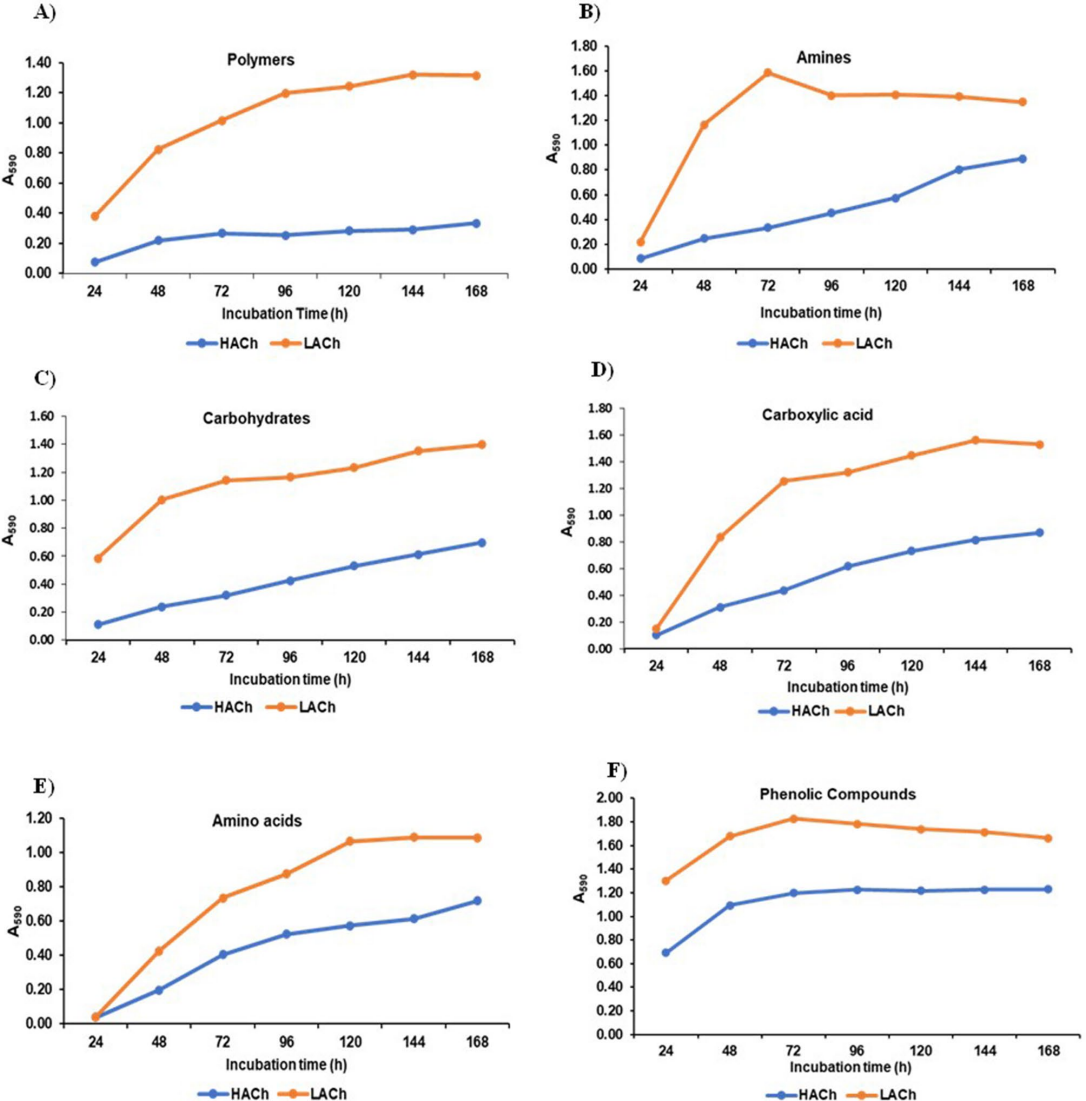
Mean optical density (A_590_) at different incubation times for utilization of six carbon source categories by gut-microbes of high altitude (HACh) and Low-altitude (LACh) chickens.

Thereafter, the effect of the substrate utilization by gut microbes was further investigated to evaluate the effect on the substrate group, where these substrates were grouped as per their functional groups as shown in Fig. 3. This data revealed that gut-microbes of high-altitude chicken used phenolic compound group more frequently from the start (i.e., 24 h), and its utilization became constant after 96 h, in contrast to the other substrate groups (carboxylic acid, amines, amino acids, carbohydrates), where utilization was prolonged at the start, and gradually increased with increasing incubation, except for the substrate polymers, which were not highly metabolized even after 168 h. On the contrary, for low-altitude chickens, gut-microbes more frequently metabolized the substrates polymers, carbohydrates, and phenolic compounds since the start, in contrast to other substrates, i.e., amines, carboxylic acids, and amino acids, which were utilized very slowly at first, and then their utilization gradually increased with increasing the incubation time, as shown in Fig. 3.

### Substrate utilization pattern

**Figure 4:**
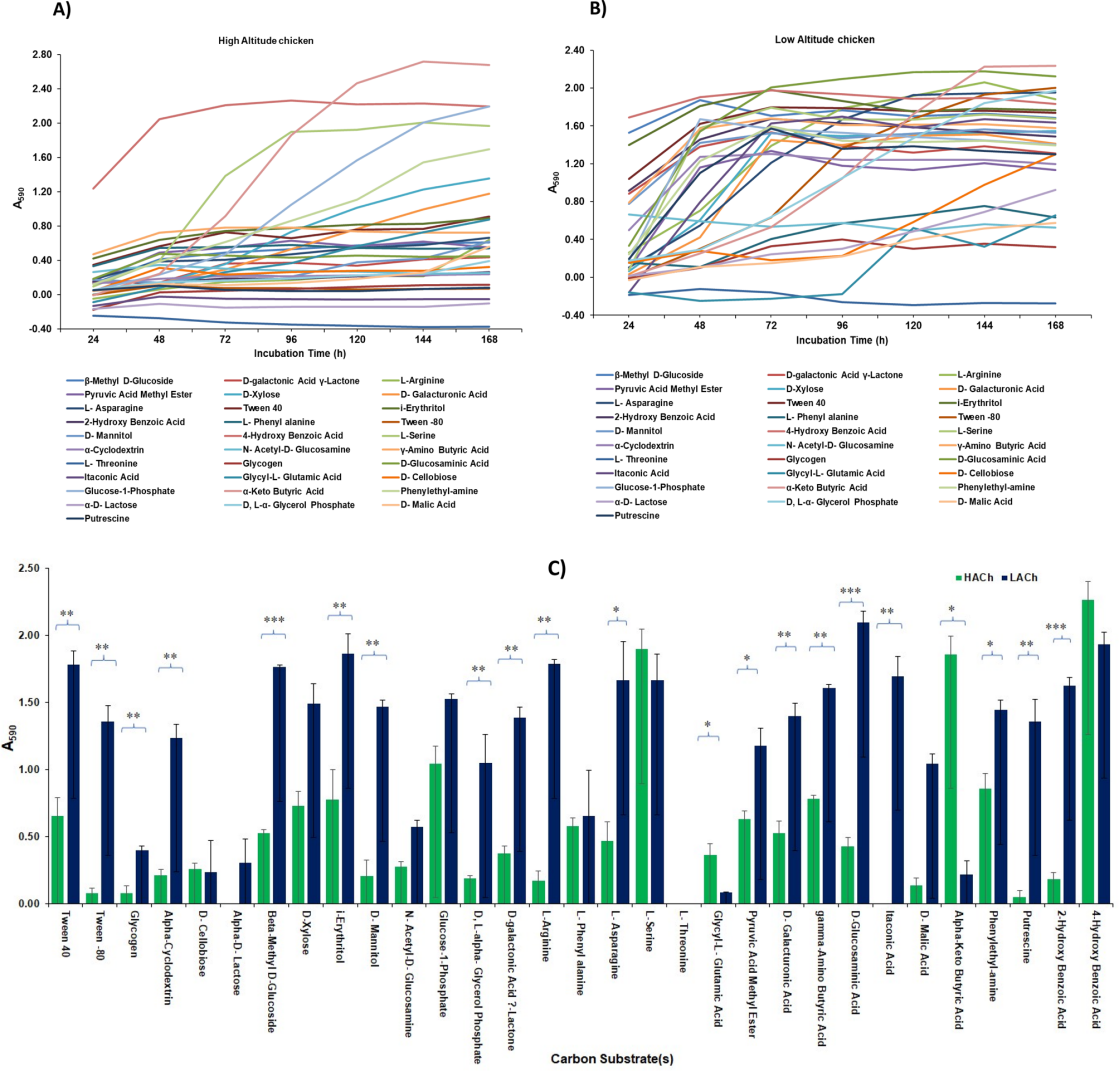
Time dependant utilization pattern of each substrate by gut-microbes of HACh (**A**) and LACh (**B**) Chicken and their comparative substrate utilization pattern (**C**). Means of the same substrate with * are significantly different. Level of significance: * : Significant (p < 0.05); **: highly significant (p < 0.01); ***: very highly significant (p < 0.001).

The utilization pattern of substrates quantified with different incubation and diversity in their utilization is shown in Fig. 4 and Table 1. Furthermore, in this study, we tried to understand the difference in the trend of utilization of each substrate among 31 carbon sources (substrates) to know the specific requirement of substrate for high and low-altitude chickens. This study provides more knowledge on the functional nature of each microorganism present in the gut of chickens reared at two different altitudes and the host-microbial interaction. The trend of utilization data revealed that utilization increased with time, then decreased after 96 h of incubation for the substrate’s Pyruvic acid, Tween-80, and Gamma-aminobutyric acid in HACh samples. In addition, for substrate “putrescine”, utilization increased with time after 48 h, gradually decreased till 120 h, and then increased up to 168 h. Based on this study, we made up two groups of substrates, one group having the same utilization pattern and the other having a differential utilization pattern during the incubation. Highly utilized substrates by high-altitude chickens were 4 hydroxybenzoic acids, alpha-keto butyric acid, l-serine, Glucose-1-phosphate, and phenylethylamine. Similarly, the least utilized substrates were threonine, Glycogen, Putrescine, alpha-d-lactose, and Itaconic acid, where no change in utilization pattern was observed after 48h as shown in Fig. 4A. Out of the 31 carbon substrates, gut-microbes of High-Altitude chickens (HACh) were intensively utilizing the substrates l-serine, 4-hydroxybutyric acid, α-keto butyric acid, and Glucose1-Phosphate (absorbance more than 1) than other substrates.

**Table 1:** Diversity in the utilization of different carbon substrates by gut-microbes of chickens reared at high and low-altitude as estimated through optical densities.

S. no.	Carbon substrate category	HACh	LACh
1.	Polymers	0.257 ± 0.079*	1.197 ± 0.156*
2.	Carbohydrates	0.439 ± 0.062*	1.16 ± 0.111*
3.	Carboxylic Acids	0.625 ± 0.128*	1.32 ± 0.128*
4.	Amino Acids	0.581 ± 0.153	0.962 ± 0.200
5.	Amines	0.455 ± 0.189*	1.401 ± 0.083*
6.	Phenolic Compounds	1.224 ± 0.469	1.78 ± 0.083

Mean with * within the same row are significantly different (p < 0.05).

Comparatively, in chickens reared in plain areas (LACh), almost all the carbon substrates *i.e.,* out of 31, 23 substrates were maximally utilized by the gut-microbes of low altitude chickens except 08 substrates, *i.e.*, Threonine, glycyl-l glutamic acid, Glycogen, d-malic acid, *N* acetyl d glucosamine, l-phenylalanine, alpha-d-lactose, and d-cellobiose. The substrate utilization was maximum at 48 h, then stagnant till 168 h. Moreover, the highly utilized substrates after 48 h are α-keto butyric acid, d-Glucosaminic acid, l-arginine, Tween-80, and 4-hydroxybenzoic acid. All the maximally utilized substrates were used at 48 h, and thereafter, their utilization pattern was stagnant, as shown in Fig. 4B.

Further, the comparative utilization pattern and the significant difference between each substrate utilization of gut-microbes from high and low-altitude chicken is graphically represented in Fig. 4C. The t-paired test results revealed a significant difference between high and low-altitude chicken’s substrate utilization at 96 h of incubation, i.e., Tween 80, Tween 40, alpha-cyclodextrin, glycogen, 2-hydroxy benzoic acid, Phenylethylamine, Putrescine, l-arginine, l-asparagine, Glycyl-l-glutamic acid, d-l-alpha glycerol phosphate, beta methyl glucoside, i-erythritol, d-mannitol, *N*-Acetyl Glucosamine, d-galactonic acid-gamma-lactone, Pyruvic acid, d-Galactouronic acid, d-glucosaminic acid, gamma-aminobutyric acid, itaconic acid, and α-keto butyric acid.

Furthermore, the data were also analysed for different groups of substrates, e.g., polymers, carbohydrates, Carboxylic acids, amino acids, amines, and phenolic compounds, which indicated significant (p < 0.05) differences in the utilization pattern between HACh and LACh (Table 1). Higher utilisation of polymers, carbohydrates, carboxylic acids, and amine group substrate was observed by LACh gut-microbes, whereas no significant difference was observed between amino acids and phenolic compounds (Table 1).

Since the microbes were known to be very sensitive to the alteration of the environment, therefore, the carbon substrate utilization of the gut microbial population was utilized to predict the functional diversity, richness, and evenness of microbial species in the chickens. Thus, the absorbance at 96 h was used to determine the shannon diversity, shannon evenness, macIntosh Diversity, macIntosh evenness and simpson diversity parameters. These diversity indices reflect the influence of altitudinal variation on the compositions and functions of gut microbes in chickens. Overall, the results revealed significant (p < 0.05) higher richness, evenness, and abundance of microbial flora in the LACh samples as compared to HACh samples (Table 2). However, no significant difference was observed on Simpson diversity index between the gut-microbes of high-versus low-altitude chicken (Table 2).

**Table 2:** Different diversity indices to determine the richness, evenness and abundance of microbes among samples at 96 h of incubation.

Diversity indices/evenness	HACh	LACh
Shannon diversity	3.005 ± 0.038*	3.27 ± 0.013*
Shannon evenness	0.905 ± 0.0052*	0.986 ± 0.005*
Macintosh diversity	0.969 ± 0.0017*	0.9508 ± 0.0042*
Macintosh evenness	0.895 ± 0.014*	0.981 ± 0.0001*
Simpson diversity	0.987 ± 0.0008	0.984 ± 0.001

Mean with * within the same row are significantly different (p < 0.05).

### Principal component analysis (PCA) of carbon substrates utilization

**Figure 5:**
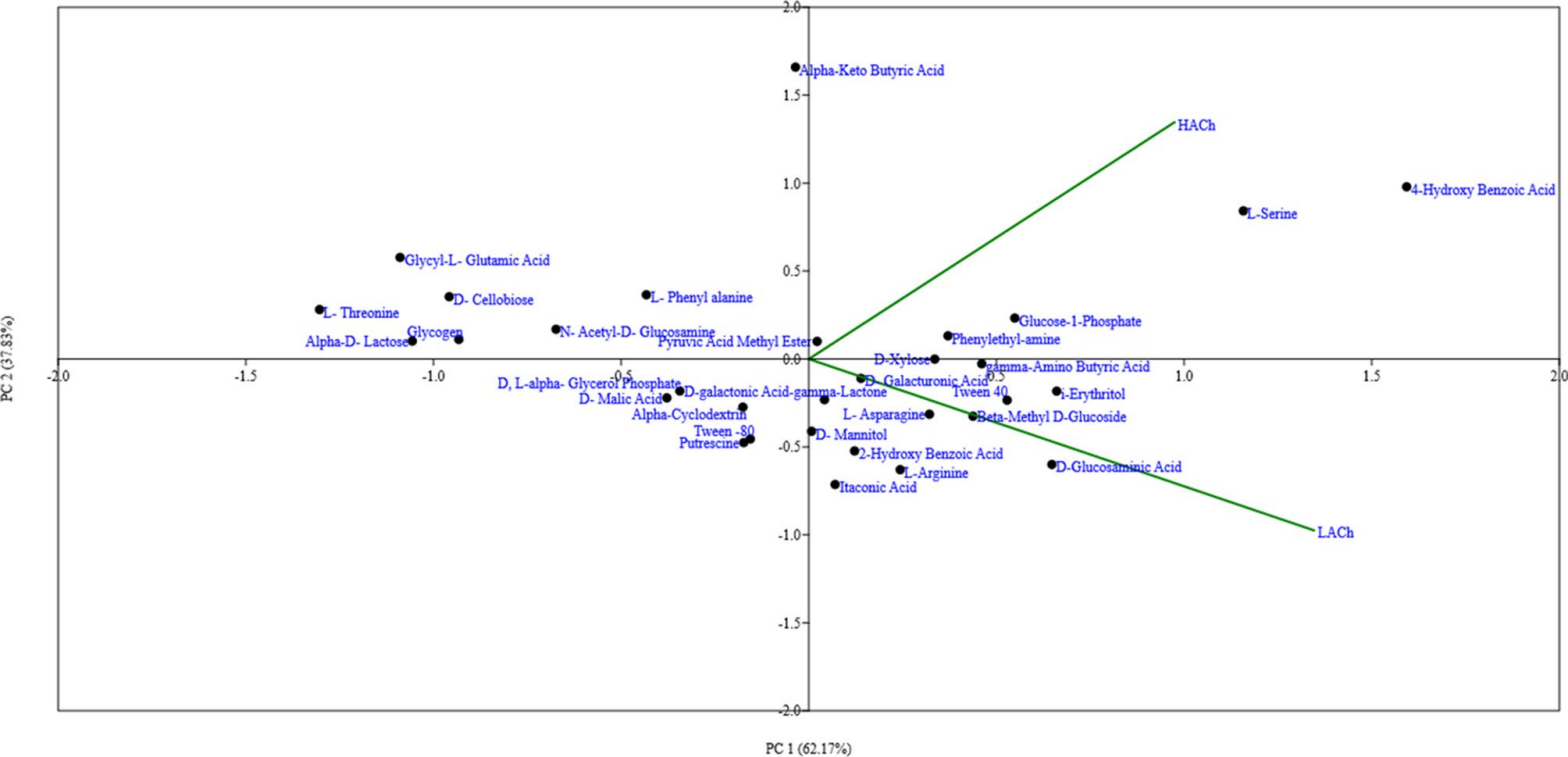
PCA analysis of HACh and LACh (PAST 4.03, 2020).

The PCA data indicated 62.17% variation in substrate utilization between the LACh and HACh chicken for first component, whereas 37.83% variance for the second component (Fig. 5).

## Discussion

Previous investigations on the microbial composition of chicken feces have revealed a substantial dominance of bacterial phyla firmicutes specifically *Lactobacillus* with a decreasing abundance of phyla proteobacteria, Actinobacteria, and Bacteroidetes^27–29^. These abundant microbes are mostly known to be facultative anaerobic in nature (could grow in the presence or absence of oxygen)^30^. Moreover, even though the bacteria can tolerate oxygen, their metabolism could be different in in-vitro environment than in the gut^31^. Hence, this study compared the metabolic potential of the facultative anaerobic gut microbes from chickens reared at different altitudes by determining their substrate utilisation pattern under aerobic conditions. Since the metabolism of various carbon sources is a vitally crucial functional characteristic of microbes, hence, this assay emphasizes the difference in functional diversity based on the variation in profile and pattern of the carbon substrates metabolized by microbes without drawing any conclusion about their function in the gut. This variation in metabolic activities could be due to the rearing altitudinal conditions of broiler chickens, where low-altitude chickens would have experienced multiple exposures to environmental microbes because high-altitude mountain areas are already reported to have two times lower relative abundance and diversity of bacterial communities in the environment than low-altitude and would have affected their metabolizing potential^32,33^. Therefore, low-altitude chickens may have utilized more substrates corresponding to their higher metabolic activity, in comparison to high-altitude animals, which less likely utilized the common carbon substrates. This observation substantiates that the gut microbes of high-altitude animals are diverse and require unique substrates for their metabolic activities. Subsequently, these deviations in carbon source utilization could relate to the difference in the functional diversity of gut microbial communities in the chicken(s) reared under different geographical conditions and variations in microbial secretary molecules.

Further, microorganisms are known to have highly regulated metabolic capabilities for efficient use of available substrates, including preferential substrate usage^34^. Indeed, the changes in environment and nutrients are reported to have a role in the acceleration and alteration of the biosynthesis and diversity of microbial metabolites^35^. Therefore, based on the existing knowledge about the specific substrate preferences of microbes and their utilization, the following interpretations have been made on the metabolic diversity of microbes and their application in industries as well as the health of the host. In the substrate category, polymers, tween-40, tween-80, α-cyclodextrin, and Glycogen, showed a significant difference in their utilization by the gut-microbes of high and low-altitude chickens. In this retrospect, the Tween-20 and 80 were already the known substrates for determining the lipase and esterase enzyme-producing microbes^36,37^. In the present study, the gut-microbes of High-altitude chickens were found to be much less utilizing the Tween-40 or Tween 80 (PS 80) than the low-altitude chickens due to the less lipase enzyme producing ability of gut-microbes of high-altitude chickens. Another substrate, alpha-cyclodextrin, was previously reported for its prebiotic benefits and for helping to alleviate or avoid metabolic illness^38^. Majorly, this substrate gets utilized by microbes through the production of the enzyme Cyclodextrinase; however, microbes lacking cyclodextrinase enzyme production ability cannot readily utilize alpha-cyclodextrin^39^. Furthermore, alpha-cyclodextrin was also found to be linked with lactate and SCFAs production, which, consequently, improve gut immunity in the animal^38,40^. Moreover, the gut-microbes of high altitude were found to be scarcely catabolizing the alpha-cyclodextrin substrate compared to the Low-altitude chickens. This is speculated to be due to the presence of microbes that do not have the potential to produce the enzyme cyclodextrinase in the sample. At last, substrate glycogen was also not readily utilized by the microbes of High-altitude chickens as in Low-altitude chickens. One possible explanation for this could be due to the difference in their substrate preferences depicting the difference in the gut-microbiota of high and low-altitude chicken.

In the substrate category of carbohydrates, a significant difference was observed in the utilization of substrates Beta-methyl-d-glucoside (also known as Beta-methyl-d-glucoside), i-Erythritol, d-mannitol, d-l-alpha-glycerol Phosphate and d-galactonic acid-gamma-lactone (also known as Galactono-gamma-lactone). The β-methyl-d-glucoside substrate was already reported to be utilized by microbes that are β**-d-**glucosidase producers^41^. In addition, substrate *i*-erythritol was already reported as a tool in the fast screening for effective strains required for either laboratory or industrial preparation of enzyme erythrulose^42,43^. However, erythritol catabolism is significantly less likely studied for their role in chickens. Thereafter, substrate d-mannitol was already known to be catabolized by microbes for production of lactic acid as a by-product. This organic acid (Lactic acid) is also known to be antimicrobial in nature, which improves the growth performance of chickens by serving as substrates in their metabolism, enhancing digestion and reducing pathogen load^44,45^. At last, the substrate, Galactono-gamma-lactone, is linked with/to ascorbic acid biosynthesis, further connected to glutathione metabolism^46^, and its feeding could help the host in coping with the oxidative stress. Consequently, in the present study, the gut-microbes of high altitude were utilising either less or slowly of all the carbohydrate substrates compared to the low altitude chickens. This observation also substantiates the difference in their preference for carbohydrate substrates and their metabolic potential.

Further, the utilization of Carboxylic acid by the gut-microbes showed a significant difference in utilization of pyruvic acid-methyl ester; d-Glucosaminic acid; d-Galacturonic acid; Gamma-aminobutyric acid; Itaconic acid and alpha-keto butyric acid. Further, Pyruvic acid is reportedly involved in various pathways for the metabolism of macronutrients i.e., carbohydrate, protein, and fat) and in generating energy through the citric acid/Krebs cycle^47^. However, not much information is available related to the effect of the biomolecule pyruvic acid-methyl ester feeding effect on the health of chickens. Further, substrate d-Glucosaminic acid was found to be highly assimilated only in low-altitude chickens, indicating a higher abundance of phyla Bacteroidetes in their gut, which contributes to the energy release, biosynthesis of propionate and anti-inflammatory properties in the host^48^. However, since this study is only focused on the metabolic potential of microbes rather than their taxonomic profile, this data could be useful for determining the industrial, microbiological, and biotechnological importance of microbes. Further, the substrate d-galacturonic acid is known to be a precursor in l-ascorbic acid and the main monomer of pectin biosynthesis in plants^49^. Galacturonic acid catabolism usually involves the action of enzymes uronate isomerase and galactarate dehydratase in microbes^50^, however, its feeding effect is not well studied in promoting health and productivity in chickens. In the present study, utilization of d-galacturonic acid was found to be slower and lesser by the gut-microbes of high altitude than the low altitude chicken. Although the role of this substrate in chicken physiology is still unknown, such microbes could have industrial applications for ascorbic acid production. Another substrate, the GABA (γ-amino-butyric-acid), is already reported to naturally occur in microorganisms, plants, and animals^51^. Indeed, GABA is reported to help in enhancing the feed conversion ratio in stressful conditions, as seen by improved gut health and growth performance in dietary GABA-supplemented chickens^52,53^. Therefore, the microbes that are utilizing less GABA could be considered more helpful for the growth performance of the chickens. In this study, microbes of high-altitude chickens were utilizing less GABA and could be considered more helpful for the growth performance of the chickens. Although this study, is only focussed on metabolic potential of microbes, further studies on taxonomic profile of microbes could be helpful to determine the beneficial isolate for host and industrial applications. At last, Itaconic substrate acid was found to be less utilized by gut-microbes of high-altitude chicken. This substrate is the microbial metabolite that activates anti-inflammatory cytokine production to modulate the host’s immunity^54^. These metabolites also enhance nutrient digestibility in chickens^55,56^. Therefore, the less utilization of Itaconic acid by the gut-microbes of high-altitude chickens is speculated to be beneficial to the chicken host. In addition, another report stated that the microbes utilizing itaconic acid are majorly pathogenic in nature^57^, since this study is only focussed on the metabolic ability rather than the taxonomic profile of microbes, this study only emphasizes about their secretary metabolites and functional potential.

Furthermore, although a significant difference was observed between the catabolism of substrate Alpha keto butyric acid, it was highly assimilated by both populations of gut-microbes of high and low-altitude chicken. Alpha keto butyric acid is known as an essential nitrogen transporter in a metabolic pathway that is further involved in the biosynthesis of many amino acids (glycine, leucine, methionine, serine, threonine, valine, isoleucine), and propionate metabolism. Therefore, these microbes are considered beneficial for the biosynthesis of biomolecules in industrial applications; however, more futuristic studies are needed to know their host-microbial interrelationship in chickens reared at respective altitudes.

In the amino acid substrates category, a significant difference was observed in the utilization of l-arginine and l-Asparagine substrate by the gut-microbes of high and low-altitude chickens. In bacteria, Arginine is metabolized through the arginase pathway (ADI pathway)^58,59^, Arginine succinyl transferase (AST) pathway, and polyamine’s (putrescine and spermidine) biosynthesis in various bacteria through the involvement of essential enzymes. In addition, the presence of arginase pathways also helps the organism survive in acidic challenges and influences the antioxidant activity for countering oxidative stress^60,61^. In this study, gut-microbes of low-altitude chickens were highly utilizing the l-arginine than the high-altitude chickens, which speculates their great potential because of their secretary enzyme producing potential such as arginase, arginine succinyl transferase, succinyl ornithine transaminase, etc., having an efficient role in industries, metabolism, and health of host.

Further, for the utilization of amino acid asparagine, the microbes produce the enzyme asparaginase to catabolize l-asparagine for fumarate production^62,63^. This enzyme is commonly found in a variety of bacterial species. Indeed, asparaginase enzyme has gained wide attention in various pharmaceutical and food industries, therefore such microbes could have scope for industrial applications. In addition, the present study also states that the gut-microbes of low-altitude chicken are highly catabolizing asparagine than high-altitude, suggesting they are highly producing the enzyme asparaginase necessary for its breakdown and could be useful for industrial applications.

Then, in the substrate category amines, a significant difference in utilization by the gut-microbes was observed for Putrescine and Phenylethylamine between the chickens. Substrate Putrescine is a typical polyamine compound metabolized by microbes into produce spermidine and is essential for cell growth, stress resistance, mammalian metabolism and lipids metabolism^64^. Additionally, it has been discovered that several bacteria break down the substrate phenylethylamine, turning it into phenylacetic acid^65,66^. This secondary metabolite aids hens in better digesting and absorbing the elements in their feed. Additionally, it is in high demand as an intermediate in the pharmaceutical and perfumesindustry^67^. In this study, the microbes of low-altitude chickens were readily utilizing the amine substrates compared to the high-altitude chickens and could have scope for industrial applications. This difference in amine utilization also substantiates the difference in carbon substrate preferences of gut-microbes sampled from high and low-altitude chicken.

At last, in the substrate category of phenolic compounds, a significant difference in the utilization of 2-hydroxy benzoic acid, commonly known as salicylic acid, was observed, where microbes from low-altitude chickens were highly metabolizing this substrate than the high-altitude. In bacteria, this substrate is utilized for the biosynthesis of catecholate siderophore in bacteria and their core structures as well as in the synthesis of an antibiotic, Promysalin^68^. Although the feeding effect of this substrate on a host is unknown, this substrate utilizing microbes may have scope for industrial applications. On the other hand, the present observation also substantiates the difference in substrate utilizing profile, substrate preferences, and the metabolic potential of gut microbes in chickens reared at different altitudes.

Overall, although most of the substrates were utilized by the low-altitude chickens’ gut-microbes, substrate keto-butyric acid and Glycyl-l-Glutamic acid were significantly utilized more by the gut-microbes of high-altitude chickens. Alpha-Keto-butyrate, a precursor in the catabolism of amino-acids, threonine, and methionine, is transformed to propionyl-CoA and plays a part in the citric cycle^69,70^. However, more is needed to know about this upsurge in using keto-butyric acid and Glycyl-l-Glutamic acid in microbes and chickens. Therefore, it is necessary to understand the metabolites, biosynthesis process and metabolic pathways of high-altitude inhabitant chickens using advanced studies in the future.

Other than the gut-microbes substrate utilization study, colour development values at the chosen incubation time were further used to calculate the indices of bacterial functional diversity. These diversity indices could provide possible insight into distinct types of heterogeneity such as richness, abundance and evenness of microbes in samples, where the Shannon index is closely related and influenced by the richness of species, while the Simpson index is based on the most common abundant species^71^. In addition, Shannon’s diversity index is also related to the number of carbon substrates that bacterial community is able to degrade, whereas Shannon’s evenness index focuses on the evenness of colour development values at chosen incubation time across all utilized substrates^72^. In contrast, the McIntosh index measures uniformity and assesses the evenness of the microbial species in samples^71,73,74^. Therefore, examining the data with more than one index is prudent for understanding the richness, evenness and abundance in microbial diversity. In the present study, the significant difference between Shannon and MacIntosh diversity indices indicated higher functional diversity, richness and evenness of gut-microbes in low-altitude chickens than in high-altitude chicken. However, no significant difference was observed in the Simpson diversity index. In this view, the present findings substantiate the variation in functional diversity of the gut-microbes through their substrate preferences, utilization pattern, richness and evenness of gut microbes in chickens reared at two different altitudes.

Statistically, the principal component analysis revealed that 13 carbon substrates constituted the first main subdivision (PC1). These included 01 polymer (Tween 40), 05 carbohydrates (Beta-methyl-d-Glucoside; d-xylose, i-erythritol; d-mannitol; d-galactonic acid-v-lactone), 02 amino acids (l-Arginine and l-Asparagine), 04 carboxylic acids (d-galactouronic acid; gamma-aminobutyric acid; d-glucosaminic acid and Itaconic acid), and 01 phenolic compound (2-hydroxy-benzoic acid), of which acid, γ-aminobutyric acid, d-glucosaminic acid, i-erythritol and tween 40 were the most relevant carbon sources that had a major effect to PC1 (≥ 0.5). In addition, 08 carbon substrates constituted the secondary major subdivision (PC2). These included 01 polymer, 03 carbohydrates (d-cellobiose; Alpha-d-Lactose and *N*-Acetyl-d-glucosaminic acid)), 03 amino acid (l-phenylalanine; L-threonine and Glycyl-l-Glutamic acid) and 01 Carboxylic acid (Alpha-ketobutyric acid), amongst which Alpha-ketobutyric acid and Glycyl-l-Glutamic acid were the most important carbon source that had important effects on to PC2 (≥ 0.5). This data also substantiates a very diverse pattern of the carbon substrate utilization profile between gut-microbes of high- and low-altitude chickens, where the carbon substrate utilization preferences of gut-microbes from High altitude chickens were found to be very different from those found in Low altitude chicken’s samples. Consequently, this has primarily contributed to the variation in the microbial community, their metabolic diversity and host-microbial relationship among the chickens reared at different altitudes.

Overall, this study offers an economically efficient approach to anticipate the functional diversity of microorganisms based on their substrate utilization. Moreover, this research could also serve as a valuable resource for investigating the altitudinal effect on gut microbial populations, their correlation with the host by employing modern -omic techniques, and its contribution to poultry industry.

## Conclusions

Present findings revealed that altitude significantly affected the substrate utilization by gut microbial communities in chickens reared at different altitudes. Overall, among the 31 substrates, gut-microbes of LACh exhibited a broader range of substrates utilisation than HACh, which discloses specific preferences for certain substrates in each group. Further, the diversity indices were also found to be more diverse in low-altitude chickens, indicating a higher richness and abundance of microbial flora. These differences in gut-microbial activity, substrate preferences, and functional diversity reflect the adaptability and functional nature of gut-microbes in reaction to environmental conditions and altitudinal variations. Consequently, the present research on the functional diversity of the gut-microbes in pooled samples could serve as reference data for exploring the potential metabolites produced and their role in improving growth, nutrient utilization, and overall health of chickens at different altitudes. Furthermore, this study also emphasizes the need for -omics (metagenomics, functional genomics and metabolomics) based research to identify the substrate preferences of gut-microbes in high-altitude chickens and their resulting metabolites.

## Data Availability

Data will be available from the corresponding author upon good scientific reason and request.

## Abbreviations

AWCD: Average well colour development
HACh: High Altitude Chicken
LACh: Low Altitude Chicken
PCA: Principal component analysis
